# The Habit of Kissing

**Published:** 1888-02

**Authors:** 


### THE HABIT OF KISSING.

Kissing is out of style. Nobody does it now but sweethearts, young children, and teachers. The first blow was struck by the medical profession about the time of the decease of Princess Alice. Ever since, the practice has been denounced, and in families where proper respect is paid to hygiene, children are cautioned against promiscuous kissing.

In society a woman is not kissed twice in a season. When an old friend is greeted and she advances with her lips the victim turns her face and the caress falls askance. Possibly the very woman who is opposed to the practice takes the initiative, but her lips never meet lips. She may kiss within a fraction of your mouth—kiss your chin, your cheek, or your forehead ; kiss your "eyelid into repose," or kiss your hair—but if she has had any training, socially, she will never kiss your mouth. The repugnance to kissing is due largely to academic training. In nearly all the famous colleges for women there is a special teacher, or doctress, in physiology ; and in the so-called oral recitations the pernicious effects of osculation are considered at great length. By way of tolerating what seems to be a necessary evil, various theories are advanced and various provisions advocated. The girl who comes from Smith College, Northampton, kisses on the oblique line that falls from the left corner of your mouth, but when kissed, is so adroit in the way she jerks her head, that the point of salutation may be found on a radius from the right of her demure little mouth. The Vassar graduate kisses more than her Smith College friend, but the chin is her choice, as you will observe in an attempt to salute her. The seniors from Wesley press their kisses high up on the face almost under the sweep of the eye-lash, and the Lake Forest and Harvard Annex maidens kiss at a point equally distant from the nose and ear.

Nothing is more dainty than the kiss of a well-bred chaperone, who, mindful of the time and trouble spent over the powder box, gently presses her lips on your hair just north of your ear. The minister's wife is another sweet soul, who knows where a kiss will do the least harm, and her favorite method is an air kiss, with a gentle pressure of her cheek to your cheek. The woman of fashion, who patronizes you, and lets you visit her while she is at her siesta, kisses you anywhere about the triangle, between the eye, ear, and hair line. She has learned long ago about the incompatibility of haste and grace, and as she advances you see her lips turn in, and simultaneous with the kiss is a thick viscous noise that sounds like the tearing of a middle-aged marshmallow drop.

Married women are more addicted to kissing than single ones, as every lady knows who goes calling, and they are the very kisses that a young woman least enjoys for the reason their lips are always wet. Kissing the face of an irregularly powdered, soapy smelling dame is about as unsavory a pastime as gathering a bouquet of dandelions. The West Side women outkiss any other division of Chicago. The South Siders kiss discreetly, and the belles and matrons along the lake shore are too cosmopolitan to waste their sweetness. Their greeting is the hand-shaking prescribed by London and endorsed by the patricians of Newport and Murray Hill. Meet a Rush or Cass street lady and she frankly proffers her easily gloved hand and shakes from the shoulder to a one-two count. Her pretty cousin on Bellevue Avenue and Lake Shore drive is more artistic, and her arm is rigid from the elbow up. Go still further north, and the carefully schooled young wife and her queenly sister shake hands, but never an inch of the arm.—*Chicago Inter-Ocean.*

